# Reply to: Field experiments show no consistent reductions in soil microbial carbon in response to warming

**DOI:** 10.1038/s41467-024-45509-3

**Published:** 2024-02-27

**Authors:** Guillaume Patoine, Nico Eisenhauer, Simone Cesarz, Helen R. P. Phillips, Xiaofeng Xu, Lihua Zhang, Carlos A. Guerra

**Affiliations:** 1 German Centre for Integrative Biodiversity Research (iDiv) Halle-Jena-Leipzig, Puschstraße 4, 04103 Leipzig, Germany; 2 Institute of Biology, Leipzig University, Puschstraße 4, 04103 Leipzig, Germany; 3 Department of Environmental Science, Saint Mary’s University, Halifax, NS Canada; 4 Department of Life Sciences, Natural History Museum, London, UK; 5 Department of Terrestrial Ecology, Netherlands Institute of Ecology (NIOO-KNAW), 6700 AB Wageningen, The Netherlands; 6 Biology Department, San Diego State University, San Diego, CA 92182 USA; 7 College of Life and Environmental Sciences, Minzu University of China, 100081 Beijing, China; 8 Institute of Biology, Martin Luther University Halle Wittenberg, Halle (Saale), Germany

**Keywords:** Carbon cycle, Geochemistry

**replying to** C.Yue et al. *Nature Communications* 10.1038/s41467-024-45508-4 (2024)

The dynamics of soil microbial carbon are complex and critically important for global carbon cycles. In our previous study^1^, we presented global trends in soil microbial carbon across time and assessed the main drivers of microbial carbon change. In the accompanying Comment, Yue et al.^2^ were able to replicate our analysis, and confirmed the robustness of a decreasing trend in soil microbial carbon using bootstrapping. However, contrary to our findings, Yue et al. argue that microbial carbon decreases are likely not caused by changes in temperature, as they found no support for this relationship in warming experiments and temporal datasets. While we appreciate the additional analyses undertaken by Yue et al.^2^, we have concerns with their approach and respond to their Comment.

Yue et al.^2^ performed a meta-analysis of warming experiments to review the effects of temperature increase on soil microbial carbon. Similar to previous meta-analyses^3,4^, they found no consistent changes in microbial carbon in experimentally warmed plots, despite numerous studies having found significant effects, highlighting again the context-dependency of the relationship between temperature and soil microbial carbon. In light of these results, we argue that it is more pertinent to identify the relevant environmental conditions that determine dissimilar trajectories. Our study showed that the relationship between temperature and soil microbial carbon is non-linear, so that sites at low mean annual temperature are more sensitive to temperature change. We suspect that only limited effects of warming could be found in the dataset, mostly due to a spatial bias, as most of the studies reviewed by Yue et al. were performed in mid-latitude sites (Fig. 1). In fact, effects of experimental warming on soil microbial carbon were previously studied in other meta-analyses spanning a larger gradient in environmental conditions^3^, which confirmed our findings of strongly context-dependent effects. Thus, Yue et al. confirm that under certain environmental conditions, warming effects on soil microbial biomass are negligible, which is not in contrast to our findings^5^ nor previous meta-studies^6,7^. Interestingly, Yue et al. chose to study the relationship between the microbial carbon response and warming magnitude using a quadratic model (see their Supplementary Fig. 2), which has little ecological reasoning. Using a linear model instead shows a significant decrease of the response with increases in warming magnitude (*p* = 0.010), which supports the role of warming as a driver or microbial carbon decrease.

**Fig. 1:**
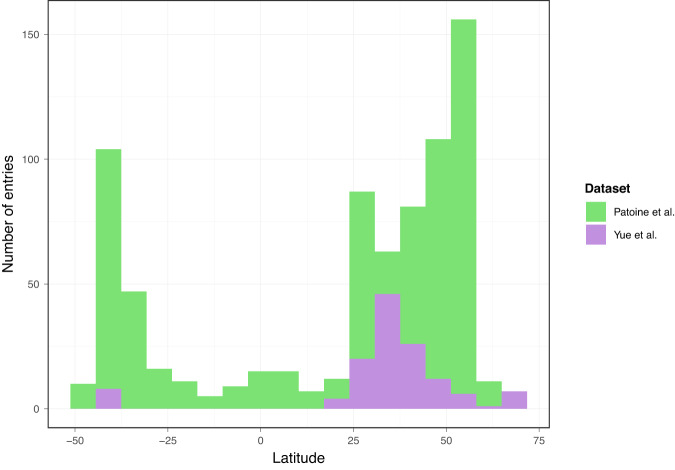
Latitudinal distributions of the datasets. Histogram comparing the latitude distribution of the dataset used by Patoine et al.^1^ with the one collected by Yue et al.^2^. The dataset by Yue et al. shows a stronger tendency toward samples in the subtropics. The two histograms are shown overlapped.

To verify how mean annual temperatures can explain variations in soil microbial carbon, Yue et al. retrieved yearly microbial carbon data from six sites, and found no significant linear relationship between yearly mean annual temperature and microbial carbon. We recognize the low availability of long-term microbial carbon data, visible in the limited extent of the dataset used by Yue et al., as four of the sites have less than 6 years of data, and the longest time series only spans 10 years. Due to the very short duration of these time series, the mean annual temperature of most sites only varied by a few degrees Celsius within a site, strongly limiting the ability of this dataset to discern any relationship. This weak statistical power is accentuated by the lack of additional relevant explanatory variables, such as vegetation and soil water content^8^. We hope that additional datasets will be available in the future to clarify these relationships.

The bootstrapping approach performed by Yue et al. confirmed the robustness of our study, as they found significant negative rates of change in microbial carbon over time in the majority of the bootstrap runs. However, Yue et al. claim that our results may be compromised by space-for-time substitution (SFT). While our study design may show aspects of SFT, this does not invalidate our findings. On the contrary, SFT is often used in ecological studies and can provide valuable mechanistic insights^9^. Especially Yang et al.^8^ found high validity in the SFT approach for modeling soil carbon processes. Most importantly, we disagree with the rationale of the approach taken by Yue et al. to test for SFT biases, and question how bootstrapping can be used for that purpose. Considering that temperature increases over time in most areas predicted, the linear relationship illustrated by Yue et al. in their Fig. 2 is to be expected, and does not indicate a sign of SFT. It is logical that subsets of the data that have a stronger negative correlation between temperature and microbial carbon are likely to predict stronger decreases in microbial carbon in a warming world.

**Fig. 2:**
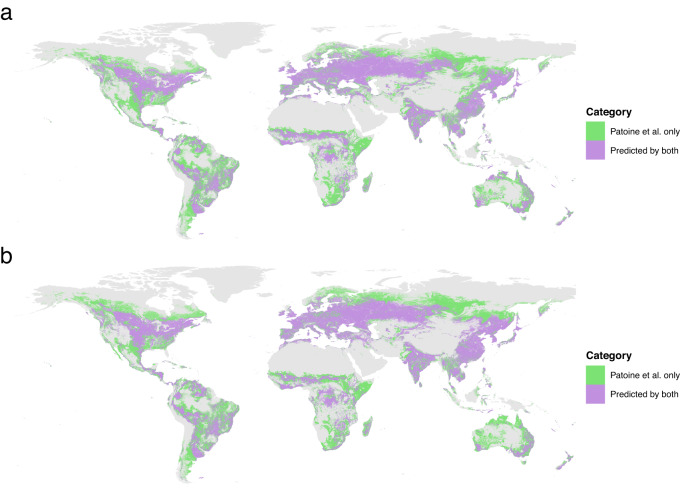
Comparison of environmental coverage analyses. Results of the unified environmental coverage analysis for 200 bootstrap random forest models simulated by Yue et al.^2^ using (**a**) the dataset from Patoine et al.^1^ (*n* = 762), and (**b**) the combined dataset including additional sites (*n* = 762 + 106). The area of applicability calculated by Patoine et al. of 2.63 M pixel locations is shown as comparison. The unified high confidence locations for Yue et al. correspond to 1.15 M and 1.14 M pixel locations, respectively for (**a**) and (**b**), equivalent to ca. 56% of the original coverage.

An additional issue in the bootstrapping approach taken by Yue et al. relates to the changes that occur to the area of applicability once the dataset is reduced. When models are trained on a subset of the data points, this changes which area of the globe can be predicted with high confidence. Using the same environmental coverage for all bootstrap models leads to making predictions in regions considered outliers by the environmental coverage analysis^10^. We note that we took a conservative approach in our original publication^11^, and recommend care in the interpretation of results outside of the area of applicability. A more appropriate approach which uses a mask made of all common areas of applicability was also performed by Yue et al. (Fig. 2), with results shown in their Supplementary Fig. 7. Using this approach, Yue et al. also found a significant mean global decrease in soil microbial carbon. It is to be noted however that their results are only valid for a smaller region of the globe, resulting in poorly comparable outcomes between the two approaches (Fig. 2).

Finally, we agree with Yue et al. that there is a gap in long-term studies on soil microbial carbon as well as its drivers across space and time, and we encourage global soil monitoring initiatives^10^, globally-distributed experimental networks, and the development of more mechanistic models^11^. However, in contrast to the approach taken by Yue et al., we highlight the urgent need to cover broad ranges in environmental conditions to fully appreciate the context-dependency of global change effects on soil microbial carbon and related processes.

## Data Availability

The input data used for this study are provided in the Supplementary Information of the original paper published in Nature Communications in July 2022.
